# Combating online health misinformation: A professional’s guide to helping the public

**DOI:** 10.29173/jchla29674

**Published:** 2023-04-01

**Authors:** Vanessa Kitchin

**Affiliations:** Knowledge Synthesis & Medical Librarian University of British Columbia Vancouver, BC, Canada

Keselman, A., Arnott Smith, C. and Amanda J. Wilson, eds. **Combating online health misinformation: A professional’s guide to helping the public**. London, U.K.: Rowman & Littlefield; 2022. Softcover: 236 pages. ISBN: 9781538162194. Price: USD$49.00. Available from: https://rowman.com/ISBN/9781538162194/Combating-Online-Health-Misinformation-A-Professional's-Guide-to-Helping-the-Public.

In an era where information is currency, the work of an information scientist has never been more critical or enmeshed with society. Many topical works cover the theme of misinformation with claims and accusations often giving those claims themselves the air of misinformation. Being that we are in an *Infodemic Era*, the convoluted nature of information access and information vetting have become, in some extreme cases, weaponized, as can be seen in a variety of recent and current events. Nevertheless, information scientists continue to serve as a beacon of truth and neutrality but the escalating danger of the spread of misinformation has left many professionals at a loss for where and how to begin resisting and preventing this phenomenon.

In *Combating online health misinformation: A professional’s guide to helping the public*, the editors compile an exemplary collection of fourteen papers addressing topics as wide-ranging as the health misinformation ecosystem, health disparities in BIPOC communities, misinformation in the clinical encounter, and the difficulty of communicating statistics. To begin the compilation, an understanding of misinformation and how it differs from disinformation is established which helps provide context and uniform understanding for the reader. The novel element to this work is that while it does cover a variety of topics related to misinformation, it hones in on combatting *online health* information, and as such is extremely relevant to the work of medical librarians.

The editors are an impressive group of medical information specialists including Dr. Alla Keselman, Senior Social Science Analyst at the National Library of Medicine (NLM); Catherine Arnott Smith, Professor, Discovery Fellow, Virtual Environments Group, Wisconsin Institutes for Discovery @ UW-Madison; and Amanda J. Wilson, NLM Deputy Associate Director for Library Operations. Dr. Keselman has held her position at NLM for the past fifteen years and is highly cited in the medical information field. Dr. Smith has a PhD in Medical Informatics and has very recent publications in the medical misinformation domain. She also teaches the Digital Health course, among other graduate level courses, at UW-Madison’s iSchool. Amanda Wilson is a formidable editor and adjunct professor at The Catholic University of America Department of Library and Information Science. She holds a MS in Library Science from the University of North Carolina at Chapel Hill. From a source perspective, the editors form an impressive, credible list of medical information and informatics professionals. With some papers written or co-written by the editors, the authority of this work is profound.

The content is organized into three parts: (1) The ecology of online health information, Susceptibility to Misinformation, (2) literacies as safeguards, and finally, (3) practice. The organization of the book and selection of relevant articles are logical, relevant and useful. In *Let the reader and viewer beware*, an article authored by one of the editors, Smith contextualizes the online nature of misinformation and clearly argues the aspects of the online environment that make this phenomenon so nuanced. She describes “the rise of the proxy” and states that “standards, codes and their progeny, checklists, and instruments, all encode shared values about information. However, these tools cannot fulfill individual users’ information needs in one critical domain that is central to understanding the misinformation problem online. That is the problem of *accuracy*”. The solution to this is the notion of consumer as evaluator. Smith further states *“*a focus on proxies is also a focus on the information consumer. Some of the various strategies to solve the problem of health misinformation put the responsibility on content creators, others on publishers, and others on trusted third parties. But still other solutions reposition patients and consumers in the center by visioning them as quality assessors. This awards control to—or places a burden on—the consumer doing the information seeking”. Her fascinating take on training information consumers to recognize proxy markers of quality in the online environment is paramount to empowering health information consumers.

Being that a plethora of articles and library research guides provide a public-facing accounting and/or suite of tools for traversing the Infodemic and medical health misinformation writ large, *Combating online health misinformation* stands out as a resource *for* medical librarians and other professionals. The usefulness of this book is that it reaches beyond the basic tenets of fact checking and understanding how information spreads, and moves towards the role of information scientist as steward; one who can offer data and insights for managing this new era. The book offers a credible source to cite and reference and the professed purpose is to “help librarians, journalists, health care professionals, community organizers, health communicators, teachers, and community health workers combat the proliferation of online health misinformation”. The insight that can be gleaned related to the BIPOC community and the danger of manipulative statistics communication, is content that is of great use to all aforementioned professionals. I highly recommend this title to any professional who works to engage science, health and information literacy in all contexts. Its depth of coverage is wide-ranging and its value is in the stellar writing and exemplary authority.

